# Rapid identification of the Asian gypsy moth and its related species based on mitochondrial DNA

**DOI:** 10.1002/ece3.3711

**Published:** 2018-01-28

**Authors:** Ying Wu, Qiuyang Du, Haiwen Qin, Juan Shi, Zhiyi Wu, Weidong Shao

**Affiliations:** ^1^ Key Laboratory for Silviculture and Conservation of Ministry of Education Beijing Forestry University Beijing China; ^2^ Zhejiang Entry‐exit Inspection and Quarantine Bureau Hangzhou China; ^3^ Zhoushan Entry‐exit Inspection and Quarantine Bureau Zhoushan China

**Keywords:** *Lymantria dispar asiatic*, *Lymantria monacha*, *Lymantria xylina*, mitochondrial DNA, specific primers

## Abstract

The gypsy moth—*Lymantria dispar* (Linnaeus)—is a worldwide forest defoliator and is of two types: the European gypsy moth and the Asian gypsy moth. Because of multiple invasions of the Asian gypsy moth, the North American Plant Protection Organization officially approved Regional Standards for Phytosanitary Measures No. 33. Accordingly, special quarantine measures have been implemented for 30 special focused ports in the epidemic areas of the Asian gypsy moth, including China, which has imposed great inconvenience on export trade. The Asian gypsy moth and its related species (i.e., *Lymantria monocha* and *Lymantria xylina*) intercepted at ports are usually at different life stages, making their identification difficult. Furthermore, Port quarantine requires speedy clearance. As such, it is difficult to identify the Asian gypsy moth and its related species only by their morphological characteristics in a speedy measure. Therefore, this study aimed to use molecular biology technology to rapidly identify the Asian gypsy moth and its related species based on the consistency of mitochondrial DNA in different life stages. We designed 10 pairs of specific primers from different fragments of the Asian gypsy moth and its related species, and their detection sensitivity met the need for rapid identification. In addition, we determined the optimal polymerase chain reaction amplification temperature of the 10 pairs of specific primers, including three pairs of specific primers for the Asian gypsy moth (*L. dispar asiatic*), four pairs of specific primers for the nun moth (*L. monocha*), and three pairs of specific primers for the casuarina moth (*L. xylina*). In conclusion, using our designed primers, direct rapid identification of the Asian gypsy moth and its related species is possible, and this advancement can help improve export trade in China.

## 1 INTRODUCTION

The gypsy moth (*Lymantria dispar* Linnaeus) is a worldwide forest defoliator, according to its geographic distribution, gypsy moth was divided into two groups of populations: the European gypsy moth and the Asian gypsy moth. The European gypsy moth originated in Europe and was introduced into the United States in 1869. It has now successfully colonized North America, destroying an estimated 12 million acres of forest annually (McManus, Schneeberger, Reardon, & Mason, 1992). This invasion has been countered by over a century of aggressive and often controversial pest control Jacques, Vince, & Kevin, 2009);. But in some cases, it just slowed but not halted the rate of spread (Sharov, Leonard, Liebhold, Roberts, & Dickerson, 2002). The Asian gypsy moth was introduced into North America in 1991 (Qian, 2000) and have arrived (but not established) in North America in recent decades; the sources of most of these moths have been ships from the Russian Far East (Animal and Plant Health Inspection Service [APHIS] 2014). As the Asian gypsy moth has a wider feeding region and stronger female flight capability than the European gypsy moth, it causes more serious harm. In 2009, the North American Plant Protection Organization (NAPPO) (NAPPO, 2009) officially approved the Regional Standards for Phytosanitary Measures No. 33, which are guidelines for cargo operations and administration of ships arriving from epidemic areas of the Asian gypsy moth. Nearly 30 ports in China have been included in the list of concerns. Members of the NAPPO, including America, Canada, and Mexico, have implemented the Regional Standards for Phytosanitary Measures No. 33 to impose control over ships arriving from the epidemic areas of Asia, and the pertinent ships are required to show proof that they do not carry the Asian gypsy moth when they arrive at the North American ports. If this proof is not provided, the ship is denied permission to directly enter the port; in addition, it incurs further anchorage inspection, fines, or even cargo withdrawal.

In addition to the Asian gypsy moth, there are two important related species, namely the nun moth (*L. monacha* L.) and the casuarina moth (*L. xylina* Swinhoe). The nun moth, commonly known as the pine needle moth, is one of the important forestry pests of pine (*Pinus* sp.) and has been found in Guangxi, Heilongjiang, and Yunnan Provinces of China (Qin & Yu, 1960). The casuarina moth mainly harms Casuarina (*Casuarina equisetifolia*), *Dimocarpus longan,* and other economic crops and is mainly distributed in Japan and India, and Fujian, Guangdong, and Taiwan in China.

To distinguish two groups of gypsy moth, several attempts were made on genetic variation of gypsy moth as well as rapid detection method (Wu et al., 2015; Stewart et al., 2016), and some methods were proven effective in the laboratory. The Asian gypsy moth and its related species intercepted at ports are usually in their egg and young larval stages. In addition, Port quarantine requires speedy clearance in the Customs Clearance Services. As such, it is difficult to identify the Asian gypsy moth and its related species only by their morphological characteristics. In recent years, molecular biology technology has become the main measure for identifying the gypsy moth and its related species at any stage, without the limitations of specimen type, insect stage, and integrity of insect morphological characteristic. Moreover, mitochondrial genes are the most widely used as molecular markers in molecular phylogeny studies, owing to their simple structure, maternal inheritance, and fast evolution rate (Miya, 2000).

Therefore, based on the mitochondrial DNA consistency in different life stages of the same species of insect, this study aimed to design specific primers that can rapidly identify the Asian gypsy moth and its related species (*L. monacha* and *L. xylina*)—using different fragments to achieve the rapid identification of these moths.

## 2 MATERIALS AND METHODS

### 2.1 Insects

Alcohol‐impregnated specimens of mature larvae of the gypsy moth (*L. dispar*) and nun moth (*L. monacha*) specimens were used, and dry specimens of adult of the casuarina moth (*L. xylina*) were used. The casuarina moths were provided by Professor Zhang Feiping from Fujian Agriculture and Forestry University (Nanping, Fujian Province, China). The nun moths were collected from Wuerqihan in the Inner Mongolia. The gypsy moth eggs were collected from Hegang in Heilongjiang Province, after rearing the eggs to the 5–6 stages of larvae to obtain samples. The feed formulation was obtained from the Northern Research Station of the US Department of Agriculture's Forest Service (Bell, Owens, Shapiro, & Tardif, 1981). Mature larvae were kept in an empty box without feed for 3 days; after the intestinal tract was emptied, the larvae were immersed in alcohol for subsequent experiments.

### 2.2 DNA isolation and polymerase chain reaction (PCR)

A total of 10 nun moths, casuarina moths, and gypsy moths were chosen. The dried specimens were completely immersed in the STE buffer contained 10 mmol/L Tris‐HCl (pH 8.0), 0.1 mol/L NaCl, and 1 mmol/L EDTA (pH 8.0) at room temperature for several hours or overnight, following which, the thoracic tissues were collected. Tris‐HCl in the STE solution provides a buffer for the DNA, leaving the DNA in a stable state and reducing the possibility of mechanical breakage. Alcohol‐soaked specimens were preserved in absolute ethyl alcohol at 4°C within 3 days before use. The specimens were cleaned by the ultrasonic cleaning device (Skymen, Shenzhen, China) for 3 min at room temperature (25°C), and 30–50 mg abdominal tissues were collected from the larvae. The TIANamp Genomic DNA Extraction Kit (Tiangen, Beijing, China) was used to extract DNA. After the DNA concentration and purity were estimated by NanoDrop 2000 (Thermo Scientific, Shanghai, China), the genomic DNA was diluted to 50 ng/μl for subsequent experiments.

General primers (Simon, Buckley, Frati, Stewart, & Beckenbach, 2006) were used for mitochondrial PCR amplification of the Asian gypsy moth, nun moth, and casuarina moth. Although the primers provided in the reference were general primers for insect mitochondrial amplification, their amplification efficiency and the base of the primer‐binding site varied from different orders. The chosen primer was the general primer for silkworm (*Bombyx mori* Linnaeus), which revised several gene loci to enhance the amplification efficiency of Lepidoptera. To improve the success rate and amplification efficiency of PCR, we modified the partial primers of the selected universal primers in this study. The specific changes made are as follows. Four complete sequences of mitochondrial DNA (accession numbers: FJ617240.1; NC_012893.1; GU994783.1; GU994784.1) were obtained from the GenBank database (https://www.ncbi.nlm.nih.gov/Genbank/). The selected universal primers were mapped with the complete sequence of the four mitochondrial DNA sequences of *Lymantria dispar* L. to find different sites on the universal primers and the complete sequences. Subsequently, to improve the success rate of amplification and amplification effect, the sites were modified to degenerate the primers; the specific primer sequence information is presented in Table 1. The PCR reaction system included 45 μl golden Mix (green) (Qingke, Beijing, China), 50 ng of template genomic DNA, and 10 μmol/L of each of the forward and reverse primers. The PCR amplification conditions were as follows: 98°C for 2 min; 30 cycles of denaturation at 98°C for 10 s, annealing at 45°C for 15 s, and extension at 72°C for 15 s/kb; and final extension at 72°C for 5 min. The PCR products were stored at 4°C. In each PCR reaction, the template DNA was replaced with sterile distilled water as a blank control. PCR products (5 μl) were electrophoresed on a 1% agarose gel for 110V, 30 min.

**Table 1:** PCR‐specific primer list

Num.	Target species		Primer sequence (5′–3′)	Optimum annealing temperature (°C)	Expected size/bp
GM‐1	Gypsy moth	F	GGGATCCAATCCTTTACCAACA	54	203
		R	GTGGTGAGCCCAAACAATAAATC		
GM‐2	Gypsy moth	F	TGGAATTACAGCTTTCCTTCTACT	54	197
		R	GGGAAATTATTCCAAATCCTGGTAA		
GM‐3	Gypsy moth	F	CCCATATTATTTCCCAAGAAAGAGG	56	152
		R	AGAGGTAAAGTAAGCTCGTGTATC		
CM‐1	Casuarina Moth	F	CATCACATTTACTCTGCCGAAATAG	56	205
		R	CGACCTCGATGTTGGATTAAGA		
CM‐2	Casuarina Moth	F	AACACTGCTCCTATAGAAAGAACA	52	210
		R	CGCTGTTCCCACTGGAATTA		
CM‐3	Casuarina Moth	F	CCTATAATAGCAAACACTGCTCCTA	52	500
		R	TTGGCCATCCTGAAGTTTACA		
NM‐1	Nun moth	F	GGAGGAGGAGATCCAATTCTTT	57	205
		R	CAGAGGTGAAATAAGCTCGAGTA		
NM‐2	Nun moth	F	TTCCCTTCATTTAGCTGGTATCTC	57	189
		R	AGAATTGGATCTCCTCCTCCA		
NM‐3	Nun moth	F	GAGCTTATTTCACCTCTGCTACT	58	200
		R	TGGCAAATACTGCTCCTATTGA		
NM‐4	Nun moth	F	ATCTTAAATCAAACCCGCCTAT	57	160
		R	CCGAAACAAATCGAACTCCT		

F, R represent the added Forward and Reverse primers, respectively, during the PCR reaction. PCR, polymerase chain reaction.

### 2.3 Sequencing for target fragments

Samples with clear and no tailing were selected and purified using TaKaRa Agarose Gel DNA Purification Kit (TaKaRa, Dalian, China). Sequencing of the target fragments was performed by the Beijing Bio‐sequencing Division of Sangon Biotech Co., Ltd (Beijing, China). The sequencing results were aligned by ClustalW Multiple Alignment program with the default value (Full Multiple alignment, Number of bootstraps = 1,000) in BioEdit software (http://www.mbio.ncsu.edu/bioedit/bioedit.html) to eliminate redundant sequences at both ends of the sequence and determine the significantly different sequences between the nun moth, casuarina moth, and Asian gypsy moth in the shared fragment, aiming to design the specific primers.

### 2.4 Design and validation of specific PCR primers

According to the sequencing results, Primer Premier 6.0 software (http://www.premierbiosoft.com/primerdesign/) was used to design and screen‐specific PCR primer pairs with a PCR product size of 150–300 bp, the primer length ranges from 15 bp to 30 bp. The specific primers were synthesized by SBS Gene Technology Co., Ltd (Beijing, China) and screened by conventional PCR. The PCR reaction conditions were the same as those mentioned in the subsection DNA isolation and polymerase chain reaction (PCR), and the optimal temperature and the minimum detection limit of specific primers were explored.

## 3 RESULTS

### 3.1 Primer specificity

We designed and screened 10 pairs of primers that could amplify the corresponding target species, as verified by PCR (Table 1). Comparison of the amplified sequences and target fragments revealed that the amplified fragment was a part of the original target fragment, indicating that these 10 primers could amplify the corresponding positional fragment of the target species. However, the primers GM‐2, GM‐3, CM‐2, and NM‐3 amplified the target species at an annealing temperature of 45°C and simultaneously amplified some of their related species. In general, during the PCR annealing process, if the primer annealing temperature increases within a certain range, the specificity of its amplification reaction increases. However, 45°C is a relatively low temperature and could result in nonspecific amplification, that is, primers annealing temperature should be fixed by concentration gradient test to achieve specific amplification. Therefore, the optimum annealing temperature of the 10 primer pairs was explored (Table 1).

**Figure 1:**
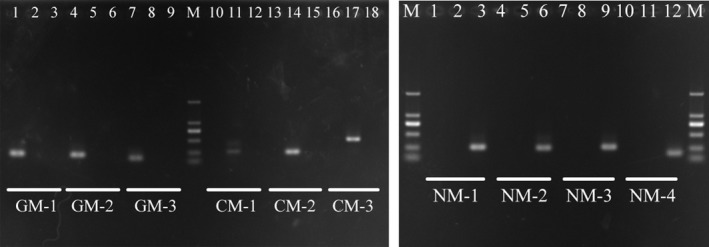
Amplification results of 10 pairs of specific primers under optimal annealing temperature conditions. M: DL2000, the template was added from left to right in each of the three lanes corresponding to each pair of primers in the gypsy moth, casuarina moth, and nun moth

Specific primers were used to amplify the sequences of the three‐moth species. As per the PCR results, the 10 pairs of primers could amplify the corresponding target species. All the 10 primers screened in this study only amplified the corresponding target species at their optimum temperature, and the other species could not be amplified (Figure 1).

### 3.2 Sensitivity according to specific primers

**Figure 2:**
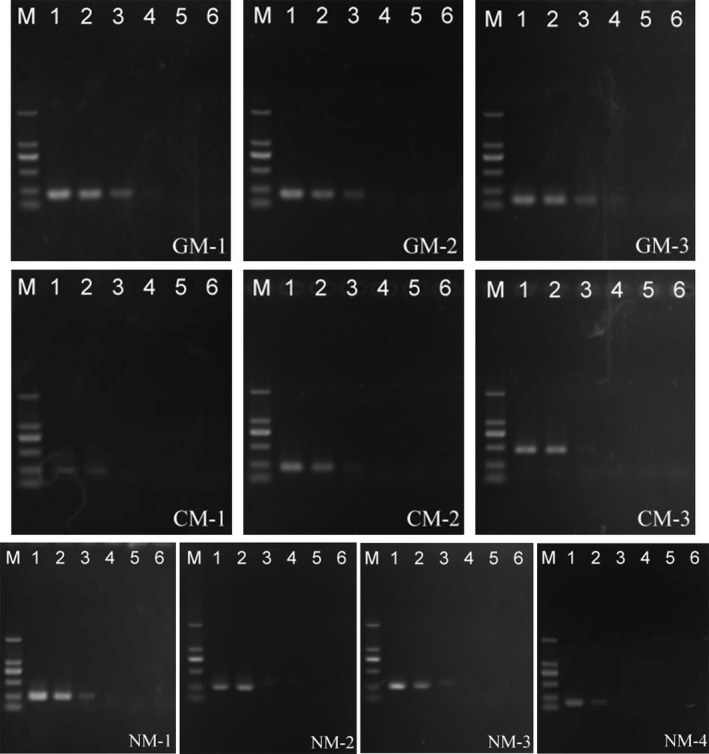
Amplification results of specific primers for three species of moths under different DNA concentrations. M: DL2000, the results of PCR amplification of 50 ng, 5 ng, 500 pg, 50 pg, 5 pg, and 500 fg templates in lanes 1–6, respectively

Six concentrations of genomic DNA (50 ng, 5 ng, 500 pg, 50 pg, 5 pg, and 500 fg) were used to determine the minimum detection limit for specific primers. GM‐3 was able to detect a minimum of 50 pg of gypsy moth DNA, whereas GM‐1 and GM‐2 only detected a minimum of 500 pg of gypsy moth DNA; furthermore, CM‐2 and CM‐3 were able to detect a minimum of 500 pg of casuarina moth DNA, whereas CM‐1 only detected a minimum of 5 ng of casuarina moth DNA (Figure 2). The lowest detectable limits of the four pairs of specific primers for the nun moth were 50 pg (NM‐1 and NM‐3) and 5 ng (NM‐2 and NM‐4).

## 4 DISCUSSION

Compared with the European gypsy moths, the Asian gypsy moths have a wider host range and a stronger flight capability; therefore, the Asian gypsy moths pose a larger threat to crops. The phytosanitary department has implemented the Regional Standard for Phytosanitary Measures No. 33 of the NAPPO since 2011 (Zhan, An, Liu, Qian, Yan, Hu, & Qiu, 2012) which has been a great inconvenience for the Sino–US ship trade and export trade in China. At present, the identification of insects in China is mainly based on morphological identification. The method is simple and convenient, but there are two obvious shortcomings: (1) the identification of eggs, larvae, and pupae is difficult and (2) fast and accurate identification is not possible for larvae at low instar (Zheng, 2012). In insects belonging to the order Lepidoptera, the adult period is short, and the egg and larval stages are relatively long, which is nonconducive to their morphological identification. In addition, the morphological classification of these insects mainly relies on the wing surface markings of adult worms that are difficult to preserve in the course of specimen acquisition and preservation, which easily leads to a loss of morphological features for recognition. Therefore, rapid molecular identification for the insects of Lepidoptera is of practical significance.

The polymerase was essential to PCR reaction, even though the primers in our study were work with the premix PCR solution named golden Mix (green) (Qingke, Beijing, China), which cannot be get easily except in China. We also tried a different polymerase TaKaRa Ex Taq^®^ DNA Polymerase (No. RR001A), and the PCR results were consistent with golden Mix (green).

As the mtDNA is organized as a circular, covalently closed, double‐stranded DNA, it provides higher stability than nuclear DNA. But during the specimens drying and DNA extraction, it also may suffer some damage. To increase the amount of DNA extracted, the dried sample was completely immersed in STE buffer for several hours to overnight before sample DNA extraction. The primary function of STE buffers is to provide a protective environment for genomic DNA, in which the DNA remains stable, ensuring maximum protection of the remaining genomic DNA from damage in dried specimens of Lepidoptera, Orthoptera, and some other insect dry specimens (Feng, Zhenning, Guigong, & Si, 2011; Zhang, Guo, & Ma, 2004; Cha, Xu, & Luo, 2006). In our study, we have tried several concentration combinations of STE buffer. The optimum concentration of ingredients of STE buffer worked with casuarina moth was 10 mmol/L Tris‐HCl (pH 8.0), 0.1 mol/L NaCl, and 1 mmol/L EDTA (pH 8.0).

Two close related species of Asian gypsy moth were used in this study, and the result showed the specific primers can easily distinguish among those species, but only one specimen collection region was used for each species. In further study, we will expand the specimen collect region among China and add species in *Lymantria* to verify the primers reliability.

In conclusion, in this study, 10 pairs of specific primers were designed and screened, including three pairs of specific primers for the Asian gypsy moth, four pairs of specific primers for the nun moth, and three pairs of specific primers for the casuarina moth. In addition, the optimal PCR amplification temperature of the 10 pairs of specific primers was determined. The size of the amplified products was consistent with the expected size, and the sequence information was accurate and reliable. Furthermore, we detected the minimum detection limits of the specific primers. Thus, the 10 specific primers designed in this study can be used to distinguish among the gypsy moth, nun moth, and casuarina moth, and its detection sensitivity meets the needs of rapid identification. Using our designed primers, direct rapid identification of the Asian gypsy moth and its related species is possible, and this advancement can help improve Port quarantine clearance speeds and eliminate the export trade obstacles in China.

## CONFLICT OF INTEREST

None declared.

## AUTHOR CONTRIBUTIONS

Juan S, Ying W, Qiuyang D, and Haiwen Q designed the research. Ying W, Qiuyang D, Haiwen Q, Zhiyi W, and Weidong S conducted laboratory work and data analyses. Juan S guided the experiment and helped perform the analysis with constructive discussions. Ying W, Qiuyang D, and Haiwen Q wrote the manuscript. All authors contributed to revisions.
